# A Hidden Recess of Atrial Tachycardia

**DOI:** 10.19102/icrm.2021.120103

**Published:** 2021-01-15

**Authors:** Aditi S. Vaishnav, Aniruddha Vyas, Yash Lokhandwala

**Affiliations:** ^1^Whitecoats Cardiac Clinic, Mumbai, India; ^2^Medanta Hospital, Indore, India; ^3^Holy Family Hospital, Mumbai, India

**Keywords:** Mapping, supraventricular tachycardia, unipolar signals

## Abstract

We present a case of regular narrow complex tachycardia in a 59-year-old woman with frequent paroxysmal palpitations, a normal electrocardiogram (ECG) in sinus rhythm, and a structurally normal heart. During electrophysiology study, a long R–P tachycardia was present at baseline, with P-waves superimposed on the T-waves and appearing to be positive in the inferior leads. Intracardiac recordings showed the atrial activation to be early in the para-Hisian region. The diagnosis of atrial tachycardia was confirmed by ventricular overdrive pacing, which showed ventriculoatrial dissociation without perturbing the atrial rate. The precise P-wave morphology was brought out in the pause, which followed rapidly delivered ventricular extrastimuli during tachycardia. Based on this information, activation mapping was conducted in the para-Hisian region, high atrial septal regions on the right and left sides, and aortic sinuses. Tachycardia was successfully ablated at one of these sites.

## Case presentation

A 59-year-old woman was evaluated by electrophysiology (EP) study for frequent episodes of palpitations and documented narrow complex tachycardia. Her sinus rhythm electrocardiogram (ECG) and echocardiogram were normal. At the beginning of the EP study, she presented in incessant tachycardia with only brief termination by overdrive atrial pacing. Intracardiac signals showed early atrial activation in the para-Hisian region.

The P-waves during tachycardia (brought about by rapidly delivered ventricular extrastimuli) were superimposed on the T-waves, narrower relative to sinus, positive in the inferior leads and V1, and negative in the aVL lead. The atrial rate remained unchanged during atrioventricular (AV) dissociation brought about by ventricular overdrive pacing, confirming the mechanism to be atrial tachycardia.

### Management

**Figure 1:**
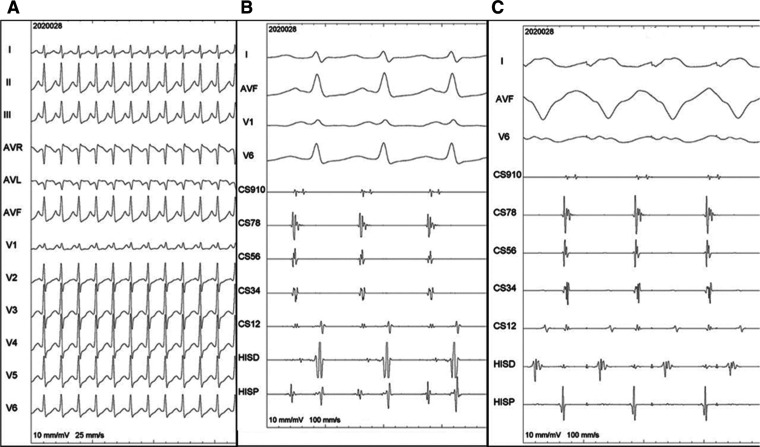
**A:** Twelve-lead ECG of long R–P narrow complex tachycardia. **B:** Intracardiac electrograms during tachycardia. **C:** Ventricular overdrive pacing during tachycardia. Shown here are surface ECG leads as well as intracardiac electrograms. CS: coronary sinus (higher numbers mean proximal electrodes); ECG: electrocardiogram; HISP/D: His bundle proximal and distal. Higher numbers mean more proximal electrodes.

**Figure 1A** shows the ECG of a long R–P narrow complex tachycardia with a cycle length of 340 ms.

**Figure 1B** shows the intracardiac signals during tachycardia with early atrial activation in the para-Hisian region. **Figure 1C** reveals that the ventricular overdrive pacing during tachycardia led to AV dissociation without a change in the atrial rate. **Figure 1** validates the mechanism to be atrial tachycardia.^1^ The morphology of the P-waves is not very clear with this, though they appear to be positive in the inferior leads and superimposed on the T-waves.

**Figure 2:**
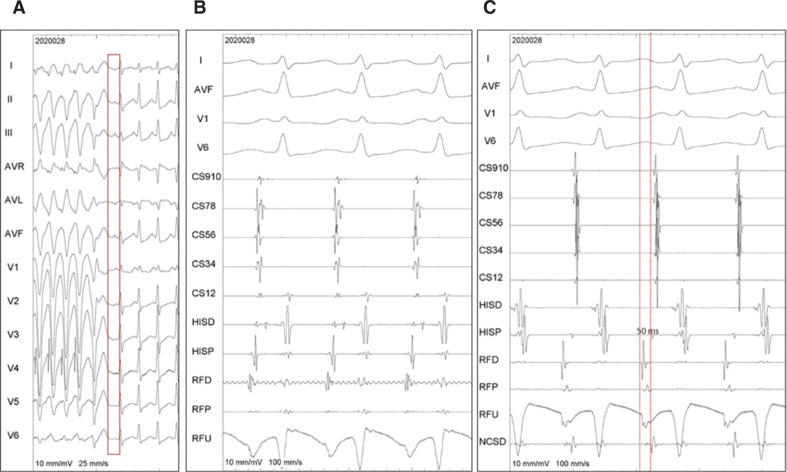
**A:** Twelve-lead ECG during rapidly delivered ventricular extrastimuli to bring out P-wave morphology (red rectangle). **B:** Signals at the noncoronary sinus as seen in the RFD/P/U channels. **C:** Signals at the site of successful ablation as seen in the RFD/P/U channels. Shown here are surface ECG leads as well as intracardiac electrograms. CS: coronary sinus (higher numbers mean proximal electrodes); ECG: electrocardiogram; HISP/D: His bundle proximal and distal; RFD/P/U ablation catheter distal, proximal, and unipolar.

**Figure 2A** demonstrates that rapidly delivered ventricular extrastimuli revealed the morphology of the P-waves. They had an inferior axis, were narrower than sinus P-waves, negative in aVL, and positive in V1. Possible sites to be mapped existed around the high interatrial septum, the perinodal region, and the aortic sinuses.

**Figure 2B** shows that the atrial signal in the noncoronary sinus occurred 36 ms earlier than the P-wave. A sharp, early negative intrinsic deflection in the unipolar signal was apparent but was preceded by a small initial R-wave; this, along with the P-wave being negative in the aVL, argue against this being the site of successful ablation.^2^ As expected, a test of radiofrequency (RF) energy here was unsuccessful.

**Figure 3:**
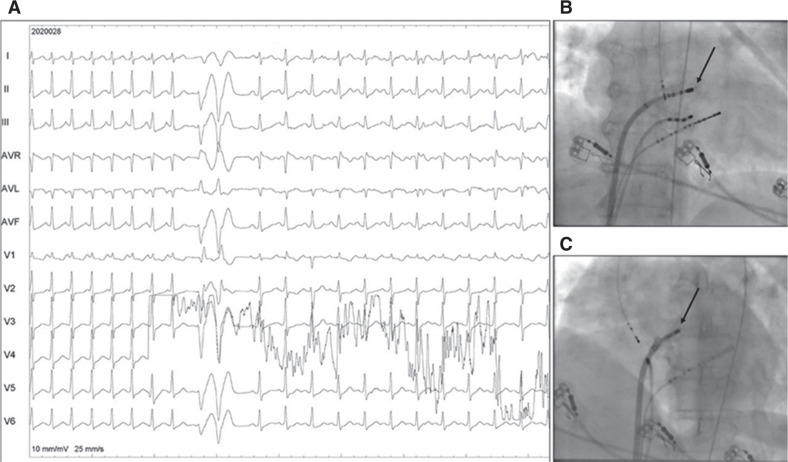
**A:** Twelve-lead ECG during radiofrequency energy delivery. **B,C:** Anteroposterior and left anterior oblique fluoroscopic views showing the ablation site (arrow).

Mapping was conducted in the right atrium near the high inter-atrial septum, the para-Hisian region, and the pulmonary veins^3^; however, no site earlier than the noncoronary sinus could be found. **Figure 2C** shows the site of successful ablation wherein the local “A” was 50 ms earlier than the P-wave and clearly earlier than the corresponding signals in the noncoronary sinus. The unipolar signal shows an early sharp negative intrinsic deflection. **Figure 3A** shows the tachycardia instantly terminating after starting RF energy (65°C and 40-W setting, via a medium-curve nonirrigation catheter), followed by a ventricular couplet and, then, sinus rhythm. Tachycardia was not inducible after this, even with isoprenaline. **Figures 3B and 3C** showing anteroposterior and left anterior oblique 40° fluoroscopic views reveal the site of successful ablation was at the mid-high left atrium, in a recess, close to the interatrial septum.
